# What factors influence a family’s decision to agree to organ donation? A critical literature review

**DOI:** 10.1080/17571472.2018.1459226

**Published:** 2018-04-23

**Authors:** Cathy Miller, Richard Breakwell

**Affiliations:** a NHS Blood and Transplant, Birmingham, UK; b College of Medical and Dental Sciences, School of Nursing, Institute of Clinical Sciences, University of Birmingham, Birmingham, UK

**Keywords:** Organ donation, brain death, family and consent

## Abstract

**Background:**There is a shortage of organs for transplantation in the UK. However, whilst 82% of the population consider donating their organs, only 35% of people have joined the NHS Organ Donor Register. Although the Human Tissue Acts (2004, 2006) and Human Transplantation (Wales) Act (2013) state that the wishes of the deceased cannot be vetoed, it is unlikely that healthcare teams will continue with the retrieval process without the family’s agreement to proceed.**Aim:**To understand what influences the decision of families to donate in order to guide clinical practice, education, training and increase donation rates to 80% in line with the NHS Blood and Transplant – Taking Organ Donation to 2020 strategy. **Method:** A literature review of published research. **Results:** Seven papers met the inclusion criteria. Several significant factors were identified that influence family decisions, including prior knowledge of the deceased’s wishes (e.g. carrying a donor card), presence at the time of the donor’s injury, understanding of brain stem death testing, ‘personal realisation’ of death and hospital related factors (e.g. information, communication and care). These were organised to form the acronym DONATE that serves as a useful mnemonic to guide the requester prior to discussing organ donation. **Conclusions:** Rates of donation of organ donation may increase through understanding family decision-making.

## Why this matters to me?

This review is important to me in helping provide an evidence base for education and training based on the knowledge and understanding of what matters to families at the end of their family member’s life regards organ donation. It is important to me that people are supported and empowered to best support potential donor families in their darkest hour.

## Background

Transplantation is the optimal treatment for many patients with end-stage organ failure. However, the demand for transplantable organs is not met by the supply of those from live or deceased donors. Currently there over 6500 people on the United Kingdom (UK) transplant waiting list; each year around 1000 people die whilst waiting for a transplant (NHS Blood and Transplant Activity report 2016–2017). In society, there is widespread support for organ donation with 8 out of 10 people saying they would want to be a donor after death. Yet, most (66% of people) never actively register to be an organ donor. In addition, around 1200 people miss out on a potentially life-saving transplant each year, due to family members overruling the decision to donate [1]. These figures draw attention to the fact that family refusals mean someone waiting for a transplant may miss their only lifesaving opportunity.

The UK’s Organ Donation Register is one avenue to formally express one’s wishes, but the current system requires individuals to opt-in to register, with 35% of the population registered to be organ donors [1]. Wales, in 2015, led the way for the UK by adopting an opt-out system of organ donation, known as presumed or deemed consent. The Act allows hospitals to presume that people aged 18 or over, who have been resident in Wales for over 12 months, wished to donate their organs at their death, unless they have objected specifically [2].

Regardless of the system in place, each year around 120 families over-rule the deceased’s expressed decision to donate (NHS Blood and Transplant, Activity report 2016/2017) [1]. Therefore, it is important to understand what influences a family’s decision to donate, as this may then inform clinical practice, educational input and future policy and help increase successful donation rates and ultimately save lives.

## Review of the literature

To explore the factors affecting donation decisions, a critical literature review was conducted, using a structured, replicable search strategy, quality appraisal process and a themed approach to data analysis.

## Search strategy

**Figure 1.**
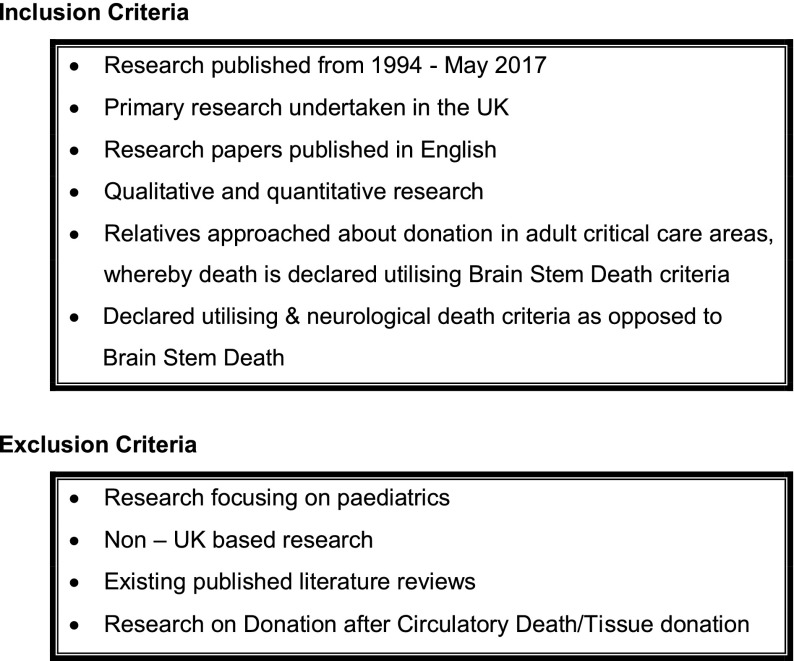
Inclusion and exclusion criteria.

An electronic search was undertaken of research papers published between the introduction of a NHS Organ Donor Register in 1994 – 2017. Five databases were searched: Medline, the Cumulative Index to Nursing and Allied Health, EMBASE, psychINFO and British Nursing Index. Search terms were: ‘organ donation’, ‘brain death’, ‘family’ and ‘consent’. Papers were limited to UK publications in English. Previous literature reviews were omitted from the initial review. The search consisted of two stages; a database search and a review of the reference lists of the identified papers. Titles, abstracts and full-text papers were reviewed for relevance and filtered using the inclusion and exclusion criteria (Figure 1). Seven papers met the selection criteria and were quality appraised prior to data collation.

## Quality assessment

Despite some concerns about the methodological quality of the located studies and potential impact on the validity and reliability of the findings and thus their transferability, due to the small number of studies, all studies were included in this review.

## Results

We undertook thematic analysis [3], to identify four main factors that influence a family’s decision-making to agree to donation for a family member.

### Family’s desire to help others

Families described choosing to donate as they found a sense of meaning at the time of donation and creating a positive outcome of a tragic situation. Participants in two studies [4,5] described honouring donation, to give meaning to the donor’s life and they wanted ‘a bit of him to go on living’. Others reported choosing to donate as it offered an alternative to switching the ventilator off [6]. The decision was influenced by the importance of quality of life, sympathy for people on dialysis and motivation by the advancement of scientific knowledge [4,5]. One study found in 14 cases when children (aged 9–18 years) were present at the time the family were approached, this impacted the decision-making process; this is an important area for further investigation [4].

### Respecting the deceased; their body, attributes and wishes

Knowing the deceased’s pre-mortem wishes significantly influenced decision-making in several studies. Participants reported the decision to donate was easier and with tangible evidence of the deceased wishes, e.g. they carried a donor card, or their wishes were known, released them from the burden of ‘making the decision’ [4–8]. Haddow highlighted the need to continue to raise public awareness, as presenting a signed donor card at the time of donation request without prior discussion with family members meant they were less confident in their decision-making [6]. In the absence of family members knowing the deceased exact wishes, knowledge of their attributes drove donation, for example relatives recounted how the deceased was ‘always doing things for others’ or was an ‘altruistic’ person [4–8].

One study examined influencing factors of non-donating families including: not wanting to let their loved one go, likening organ donation to a ‘sacrifice’. Protecting the deceased’s body was important – a fear of the body being cut and disfigured, and the thought that the deceased had suffered enough. Disfigurement of the body was cited as predictor of unwillingness to donate, despite the relative’s own positive wishes or supporting donor card [9].

### Understanding neurological death testing and organ donation

Neurological death is defined as ‘irreversible loss of capacity for consciousness combined with the irreversible loss of the capacity to breath’ [10]. It is a pre-requisite for donation, although to families the body still appears to have life [4]. In one study, 16 respondents (70%) expressed that they made their own judgement as to when their relative had died before formal neurological death testing to confirm death [6], only one study showing clear understanding of the purpose by families [11]. Three studies mentioned family members witnessing the second set of neurological death tests to be sure their loved one was dead before agreeing to donation [4,5,7].

Processing the enormity of this information about neurological death at an acutely emotional time may account for the reasons why families decline donation.

Whilst some families were well-informed about organ donation, others felt they were not. Five studies showed benefit from adjuncts to help families understand neurological death tests, including Computerised Tomography scans, explanatory leaflets, anatomical models, and allowing relatives to observe the testing process [4,5,9,11]. One study noted that 5 out of thirteen families did not take up the offer to observe [11]. It was expressed that information needs to be correct and presented in ways that are responsive to family needs [4]. Some families reported lack of information to support their understanding and decision-making [6–9]. Other families felt they were not given time to ask questions and lacked knowledge about what the process would involve [4].

One study found retrospective regret for some non-donor families, whilst no donating families regretted their decision [11]. This reinforces the need for appropriate and timely information, with time given for families to understand and absorb information.

### Professional compassionate care, language and communication skills

Excellent communication skills are key to helping families come to terms with death, noting distressing situations such as delayed diagnosis of death and ‘switching the ventilator off’ [6]. A sensitive and empathetic manner for how families were approached and early, open and honest discussions was a discriminator between the decision to donate or not [4–6,9,11]. Non-donating families reported that poor communication and language negatively influenced their decision with words such as ‘harvesting’ causing anxiety, distress and negative connotations [4,6,7]. How the news is broken to the family and timing of the donation request is a matter of professional judgment, indicating the need for training in this area.

In a longitudinal study of 46 families, 45 were positive about being approached by staff in normal clothing. Appearance of professionals mattered; families described finding it easier to talk to transplant co-ordinators (now known as Specialist Nurse-Organ Donation) who were not in uniform and one non-donating family member decided not to donate based on the consultant’s appearance in operating attire [4,6]. Furthermore, perceived poor or inadequate care prior to intensive care admission led to a donation refusal, with one pro-donation family ultimately declining donation due to stressful events [4].

## Discussion

Health care professionals need a high level of skill to navigate families through the complex and emotive journey if they are to result in both supporting families through grief and consenting to organ donation.

This paper summarises the four key findings from UK research about factors that influence the decisions of family members to agree to donate. We have re-presented these factors as a mnemonic – ‘DONATE’ to help staff involved in organ donation.

**D – Drivers** of family for donation

Drivers include:

• Family’s desire to help others and see, the deceased’s legacy continuing to exist through donation

**O – Optimal** communication

Factors includes:

• Families require clear, uncomplicated and accessible information in small and digestible pieces, about the cause and diagnosis of neurological death and decision-making support, including time to think, reflect and have questions answered [9]
• Information on the benefits of organ donation, reassurance that the donor will receive high quality of care and respectful treatment of the deceased body
• Delivered by compassionate, skilled and trusted professionals

**N – Needs at the time of donation conversations**

• Professionals caring for family members should understand and address a family’s individual needs to enable decision-making and when necessary, allowing families more time and inviting them to reconsider their initial refusal, may lead to a more enduring decision and possibly increase consent
• A service strategy should be in place to support families
• Transplant coordinator (now known as Specialist Nurse-Organ Donation), donating families were unanimously positive about their experience with the coordinator and this contact is associated with consent to donation [4]

**A – Altruism**

• Knowing the altruistic wishes of the potential donor, e.g. whether they were registered on the organ donor register or had previously expressed a wish to donate, encourage family discussion and influenced decision-making, as did the perception of the donor having altruistic values in their lifetime [4–8]

**T – Timing**

Timing factors:

• Families present at the patient’s time of injury e.g. collapse or traumatic accident were more likely to be accepting of the situation and agree to donation than those not present [8]
• Timing of donation conversation e.g. before or after outcome of the neurological death tests
• Length of time the patient was ventilated
• No ideal timing was identified for every family – family views were unique

**E – Empathy** and respect

• Caring for relatives and enabling them to make decisions about donation that are right for them requires professionals to work together to understand the ‘emotional landscape’ imposed on the family by sudden death [4]
• The type of attire worn at the time of donation conversations influences the outcome
• Positive memories of care, remain positive, whilst negative memories remain negative – despite time passing, the effect of initial care impacts upon subsequent grief [4]

The themes resonate with an international systematic review into modifiable factors influencing relatives’ decision to offer organ donation [12]. In addition, a systematic integrative literature review enhances understanding of the factors influencing bereaved families’ decisions to agree to, or decline, donation, by categorising themes into past, present and future [13]. A further literature review [14] focusing on what the diagnosis of brain death means to family members, concurs that understanding neurological death is crucial in increasing organ donation rates.

## Evaluation and further research

Bereaved families report uncertainty about death and the donation process, experiencing emotional and cognitive overload and decisional conflict, and yet they can derive emotional benefit from the altruistic act of donation. Education therefore needs to focus on two key areas:

(1) How to help families facing donation to gain understanding of definitions and processes, address anxieties and insecurities in decision-making, and to gain a sense of closure.
(2) How to guide professionals to understand the potential importance of tailoring their communication to the needs of the family. This will include their communication styles and skills as well as providing clear, accessible information given in timely and manageable pieces, allowing time for families to reflect in between.

Further research is needed to explore the decision not to donate. Non-donor families are of interest, as this area is to date not very well explored and may serve to yield a wealth of knowledge and increased understanding to what negatively influences family decisions. The impact of religion, age, culture and race has on the decision-making process is not explored within this paper. If there is to be better understanding about organ donation across diverse groups, to minimise inequalities, this is a key area for exploration.

## Conclusion

Transplant waiting lists are ever growing in the UK and family consent remains one of the most crucial factors to the success of organ donation programmes. This paper provides a review of UK evidence and key themes are aligned to a framework DONATE, to help structure support provided by health care professionals liaising with families. The DONATE framework aids consideration of drivers affecting family decision-making; ensuring optimum communication and explanation of neurological death tests whilst meeting the unique needs of families and listening out for altruistic values of the deceased. It is important that conversations are timed to meet the relative’s needs and that the discussion is delivered with genuine empathy as this conversation lives on with the family. The request process takes time, involves collaboration and expertise to meet the family’s needs. As organ donation is a time limited, once only option, involvement of specialist nurses trained in organ donation can encourage family discussions and help identify and meet the family’s needs [4]. DONATE aims to optimise effective communication and personalised decision-making for families and this raises the need to ensure that professionals are skilled communicators to truly make a difference to organ donation rates.

## Disclosure statement

The lead author’s role as the Head of Education and Professional Development, NHS Blood and Transplant, may suggest a conflict of interest. Therefore, the second author provides independent scrutiny.

## Funding

The study was carried out as part of an MSc in Health Studies, supported by NHS Blood and Transplant and University Hospitals Birmingham NHS Foundation Trust.
